# Bone Scan Index: a prognostic imaging biomarker for high-risk prostate cancer patients receiving primary hormonal therapy

**DOI:** 10.1186/2191-219X-3-9

**Published:** 2013-02-06

**Authors:** Reza Kaboteh, Jan-Erik Damber, Peter Gjertsson, Peter Höglund, Milan Lomsky, Mattias Ohlsson, Lars Edenbrandt

**Affiliations:** 1 Department of Molecular and Clinical Medicine, Clinical Physiology, Sahlgrenska University Hospital, Sahlgrenska Academy at the University of Gothenburg, Gothenburg, 413 45, Sweden; 2 Department of Urology, Institute of Clinical Sciences, Gothenburg University, Gothenburg, 413 45, Sweden; 3 Competence Centre for Clinical Research, Lund University Hospital, Lund, 221 00, Sweden; 4 Department of Theoretical Physics, Lund University, Lund, 221 00, Sweden; 5 Department of Clinical Sciences, Malmö, Lund University, Lund, 221 00, Sweden

**Keywords:** Image analysis, Radionuclide imaging, Bone metastases, Prostate cancer

## Abstract

**Background:**

The objective of this study was to explore the prognostic value of the Bone Scan Index (BSI) obtained at the time of diagnosis in a group of high-risk prostate cancer patients receiving primary hormonal therapy.

**Methods:**

This was a retrospective study based on 130 consecutive prostate cancer patients at high risk, based on clinical stage (T2c/T3/T4), Gleason score (8 to 10) and prostate-specific antigen (PSA) (> 20 ng/mL), who had undergone whole-body bone scans < 3 months after diagnosis and who received primary hormonal therapy. BSI was calculated using an automated method. Cox proportional-hazards regression models were used to investigate the association between clinical stage, Gleason score, PSA, BSI and survival. Discrimination between prognostic models was assessed using the concordance index (C-index).

**Results:**

In a multivariate analysis, Gleason score (*p* = 0.01) and BSI (*p* < 0.001) were associated with survival, but clinical stage (*p* = 0.29) and PSA (*p* = 0.57) were not prognostic. The C-index increased from 0.66 to 0.71 when adding BSI to a model including clinical stage, Gleason score and PSA. The 5-year probability of survival was 55% for patients without metastases, 42% for patients with BSI < 1, 31% for patients with BSI = 1 to 5, and 0% for patients with BSI > 5.

**Conclusions:**

BSI can be used as a complement to PSA to risk-stratify high-risk prostate cancer patients at the time of diagnosis. This imaging biomarker, reflecting the extent of metastatic disease, can be of value both in clinical trials and in patient management when deciding on treatment.

## Background

Life expectancy is a major factor to be considered in the management of prostate cancer patients. Risk stratification schemes based on clinical T stage, Gleason score and prostate-specific antigen (PSA) are widely used to estimate risk in individual patients. The extent of bone metastases is also associated with survival [1,2], but there has not been any clinically useful technique of quantifying the skeletal tumour burden and including this information in the risk assessment. Bone scintigraphy, however, is commonly used to assess skeletal tumour burden in prostate cancer patients, both in clinical routine and in nearly every clinical trial. In order to extract as much clinical information as possible from the bone scans, the Bone Scan Index (BSI) was developed as a quantitative tool to improve the interpretability and clinical relevance of the bone scan [3]. BSI is a method of expressing the tumour burden in the bone as a percentage of the total skeletal mass.

BSI has recently been presented as a response indicator in patients with castration-resistant metastatic prostate cancer who have received chemotherapy [4]. The patients had bone scans at baseline and at 3-month and 6-month follow-up, and BSI changes post-treatment were a significant prognostic factor for survival. A doubling of BSI post-treatment resulted in a 1.9-fold increase in the risk of death. These results showed the feasibility of capturing bone scintigraphy data as a single quantitative measure and thereby allowing bone scans to be explored as imaging biomarkers. Furthermore, changes in BSI post-treatment were significantly associated with survival, but post-treatment changes in PSA were not, while adjusting for changes in BSI.

In order to improve the usefulness of BSI, we recently presented a fully automated method of quantifying BSI [5]. The automated method was evaluated in a group of newly diagnosed prostate cancer patients, and BSI increased the predictive accuracy for death as a result of prostate cancer when added to a model containing pre-treatment clinical stage, Gleason score and PSA. The 5-year probability of dying as a result of prostate cancer was under 6% for patients with BSI < 0.1 and over 78% for patients with BSI > 1.0 at the time of diagnosis. Initial treatment may influence outcomes, and that was not adjusted for in our previous study. Furthermore, low- and intermediate-risk patients (median PSA = 16.8 ng/mL) were included, and many of these patients probably had a specific indication for receiving a bone scan, for example, bone pain. This may have resulted in a selection bias. High-risk patients at the time of diagnosis have been referred for bone scans before treatment decisions as part of the clinical routine. The purpose of this study was to explore the prognostic value of BSI obtained at the time of diagnosis in a group of high-risk prostate cancer patients receiving primary hormonal therapy.

## Methods

### Patients

All patients with the diagnosis of prostate cancer who, during the period 2002 to 2008, had undergone a whole-body bone scan < 3 months from the time of diagnosis at the Sahlgrenska University Hospital, Gothenburg, Sweden, were retrospectively considered for inclusion in the study. Only patients at high risk who received primary hormonal therapy were included (chemical or surgical castration (*n* = 97), anti-androgen deprivation as monotherapy (*n* = 13), total androgen blockade (*n* = 11), neoadjuvant treatment before curative treatment (*n* = 6), a combination of external irradiation with anti-androgen deprivation (*n* = 2), radical prostatectomy (*n* = 1)). None of the patients had received any type of PCa treatment prior to the bone scan. We defined patients as high-risk if at least one of the following criteria was met [6]:

• T2c/T3/T4

• Gleason score 8 to 10

• PSA level > 20 ng/mL

In total, 130 patients were included in our analysis. Patient characteristics are presented in Table 1. During the study period, patients at high risk at the time of diagnosis were referred for bone scan examination before treatment decisions as part of the clinical routine. The study was approved by the Research Ethics Committee at Gothenburg University.

**Table 1:** **Patient characteristics (*n *= 130)**

**Patient characteristic**	**Value**
Age (years), median (IQR)	76.0 (69 to 84)
BSI	
BSI = 0 (M0), no. (%)	72 (55)
BSI > 0 (M1), no. (%)	58 (45)
BSI > 0 (M1), median (IQR)	1.7 (0.7 to 7.4)
PSA (ng/mL), median (IQR)	59 (23 to 232)
Clinical T stage (*n* = 129)	
T1, no. (%)	19 (15)
T2, no. (%)	29 (22)
T3, no. (%)	54 (42)
T4, no. (%)	27 (21)
Gleason score (*n* = 127)	
6, no. (%)	6 (5)
7, no. (%)	47 (37)
8, no. (%)	30 (24)
9, no. (%)	35 (28)
10, no. (%)	9 (7)

BSI, Bone Scan Index; IQR, interquartile range; PSA, prostate-specific antigen; M, M category in staging system for prostate cancer spreading to distant parts of the body.

### Data collection

Overall survival was defined as the time from bone scan examination to death from any cause. Survival data and PSA values collected from the computerised medical records were updated until 24 September 2010. A total of 76 were dead at follow-up with a median survival time of 2.7 years (IQR 1.6 to 4.2) and 54 were alive with a median follow-up time of 5.1 years (IQR 3.9 to 8.5). Data on clinical T stage and Gleason score at the time of diagnosis were collected from the Swedish National Cancer Registry [7].

The percentage of the skeleton affected by tumour mass in a bone scan was measured by calculating the BSI. We have recently presented an automated method [5] based on the clinically validated methodology for manually computing BSI as presented by a group at the Memorial Sloan-Kettering Cancer Center in New York [3,8,9]. The automated method is trained to mimic an expert reader in distinguishing hotspots due to metastases from those caused by factors such as degenerative disease or fractures. Manual correction was required in less than 5% of the patients to exclude hotspots clearly misclassified and representing features such as a very large urinary bladder, a urinary catheter attached to a drainage bag or urine contamination. No other manual intervention was applied. The method is implemented in the commercially available software package EXINI bone™ (EXINI Diagnostics AB, Lund, Sweden).

### Statistical methods

Cox proportional-hazards regression models were used to investigate the association between clinical stage, Gleason score, PSA, BSI and survival, both in univariate and multivariable modelling. Hazard ratios were estimated from the Cox models together with 95% confidence intervals. The concordance index (C-index) was used to discriminate between the different survival models [10]. The significance of a difference in C-index between different models was calculated using the method described by Haibe-Kains et al. [11].

Kaplan-Meier estimates of the survival function were used together with the log-rank test to indicate a significant difference between four groups of patients: without metastases (M0), with BSI < 1, BSI = 1 to 5 and BSI > 5. The BSI levels 1 and 5 were used to categorize the patients since that resulted in approximately the same number of patients in each of the metastatic groups.

The survival rate expected in a group of men in the general population with the same age as the M0 group was calculated. This control survival curve of the general Swedish population was generated using the Hakulinen method [12], using data collected from the administrative agency Statistics Sweden. This group is similar to the M0 group with respect to other possible factors affecting survival, except for the patients of the M0 group having prostate cancer.

In the survival analysis, data were censored at a follow-up time of 5 years. All analyses were carried out using the R statistical computing environment.

## Results

**Figure 1:**
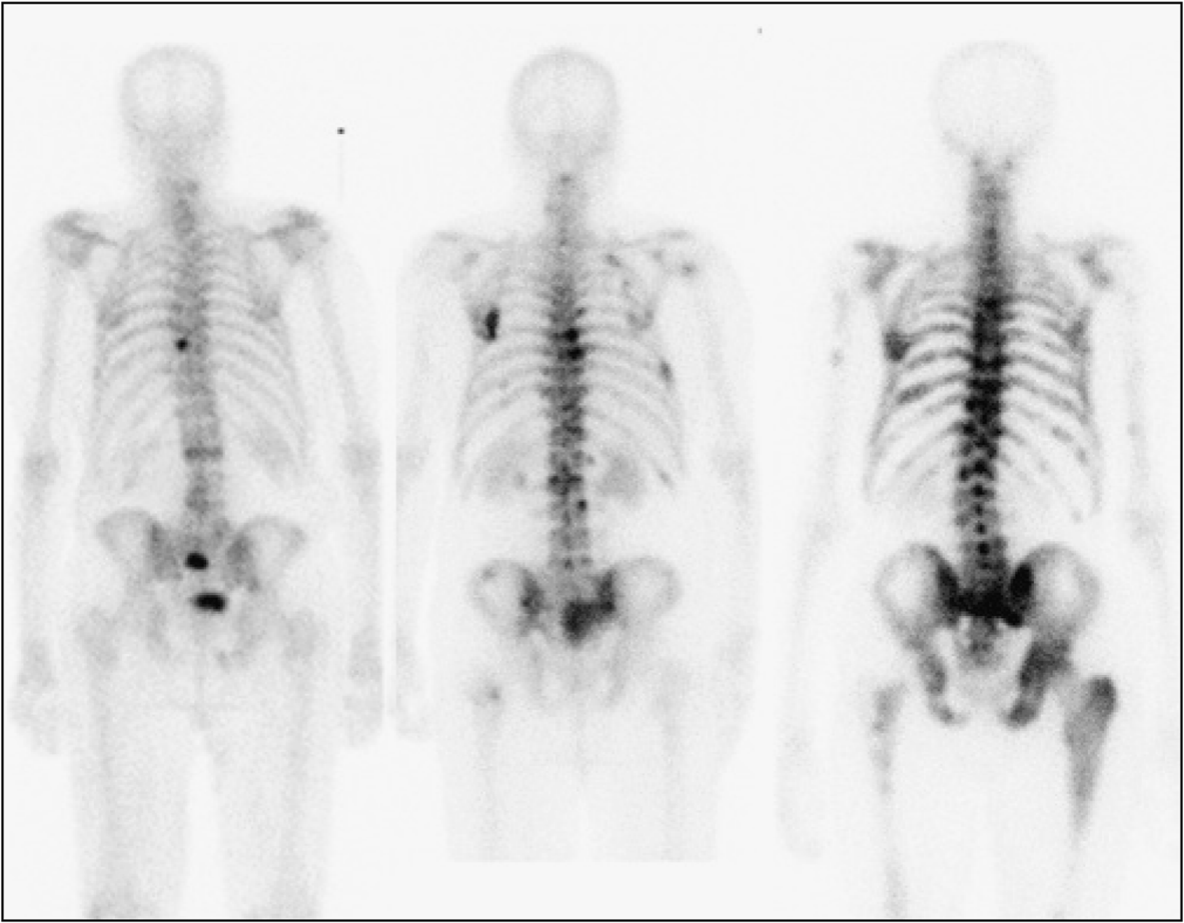
**Three patients, one each from the groups BSI < 1, BSI = 1 to 5 and BSI > 5.** The actual BSI values were 0.7, 2.2 and 8.1, respectively.

**Figure 2:**
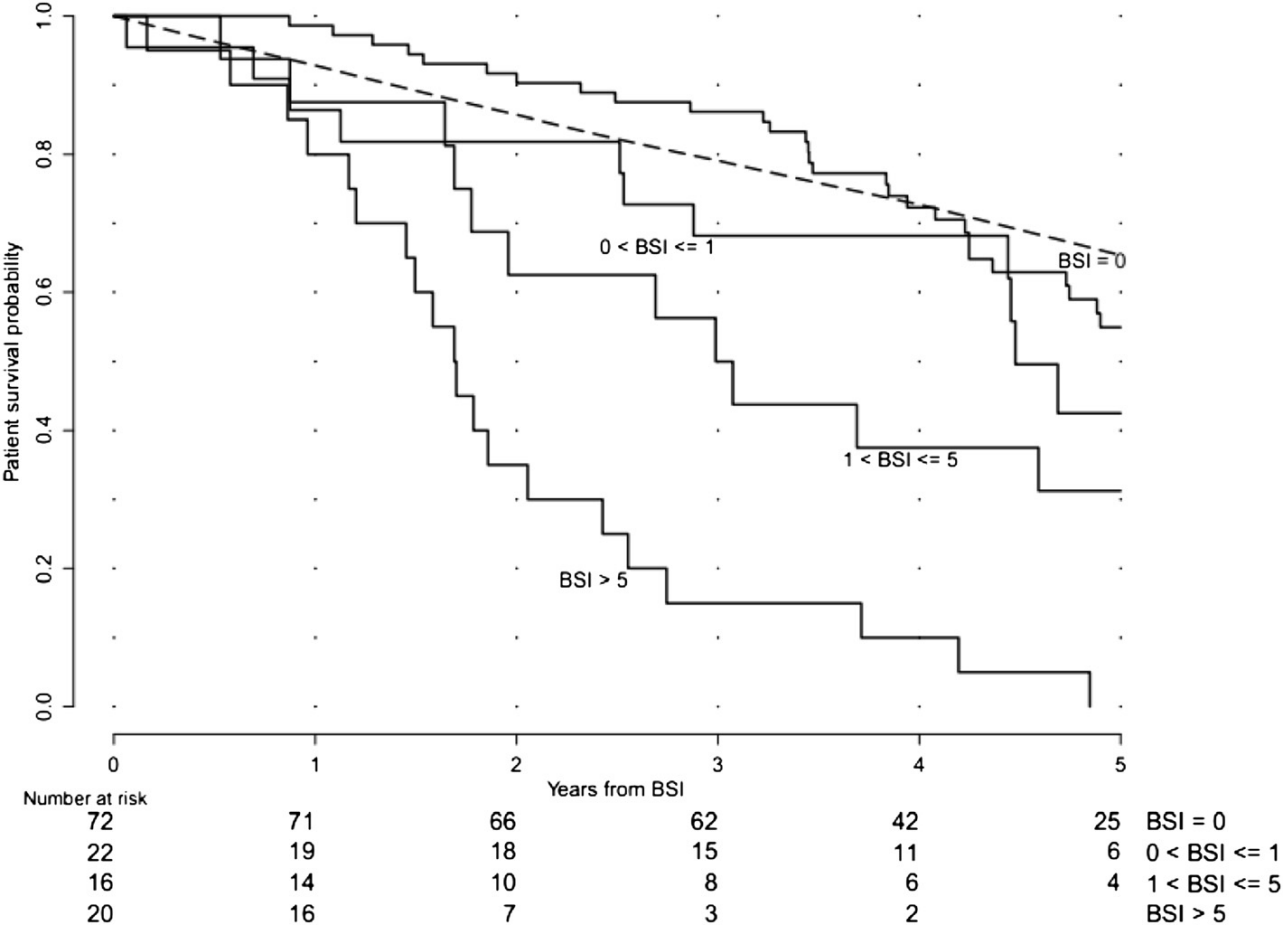
**Kaplan-Meier curves showing patient-survival probability stratified by BSI categories.** The difference between the four groups of patients without metastases (M0) and with metastases BSI < 1, BSI = 1 to 5 and BSI > 5 was statistically significant (*p* < 0.001). The broken line shows an age-matched control survival curve for the M0 group.

The 5-year probability of survival was 41% in the total group of high-risk patients. Metastatic disease was present in 58/130 (45%) of the patients. The 5-year survival probabilities were 24% and 55% in patients with (M1) and without (M0) metastases, respectively. Considering the tumour burden, measured using the continuous variable BSI, and not only the presence of metastases (M0 or M1), it was possible to divide the metastatic group. We divided this group into three subgroups in order to have approximately 20 patients in each subgroup: BSI < 1 (*n* = 22), BSI = 1 to 5 (*n* = 16) and BSI > 5 (*n* = 20). Figure 1 shows one patient from each of these subgroups illustrating the extent of disease. The Kaplan-Meier curves for patients M0, BSI < 1, BSI = 1 to 5 and BSI > 5 were significantly different (*p* < 0.001), and the 5-year survival probabilities were 55%, 42%, 31% and 0%, respectively. The survival curve for the M0 patients is above the corresponding survival curve for the general male population of the same age for the first 4 years (Figure 2). The corresponding value for an age-matched control cohort was 65%.

In the univariate analysis clinical stage, Gleason score, PSA and BSI were all associated with survival (Table 2). In a multivariate analysis, Gleason score (*p* = 0.01) and BSI (*p* < 0.001) were associated with survival, but clinical stage (*p* = 0.29) and PSA (*p* = 0.57) were not prognostic. The C-index increased from 0.66 to 0.71 (*p* = 0.006) when adding BSI to a model including clinical stage, Gleason score and PSA.

**Table 2:** Survival analysis demonstrating association between clinical stage, Gleason score, PSA, BSI and survival

**Variable**	**No. of patients**	**Hazard ratio**	***P *value**
Univariate analysis			
Clinical T stage	129	1.53 (1.17 to 1.99)	0.002
Gleason score	127	1.39 (1.10 to 1.76)	0.006
PSA	130	1.0003 (1.0001 to 1.0005)	0.001
BSI	130	1.21 (1.14 to 1.28)	< 0.001
Multivariate analysis			
Clinical T stage	126	1.34 (1.02 to 1.77)	0.04
Gleason score	126	1.36 (1.07 to 1.73)	0.01
PSA	126	1.0003 (1.0001 to 1.0005)	0.006
Multivariate analysis			
Clinical T stage	126	1.17 (0.87 to 1.57)	0.29
Gleason score	126	1.37 (1.07 to 1.75)	0.01
PSA	126	1.0000 (0.9998 to 1.0003)	0.57
BSI	126	1.19 (1.12 to 1.28)	< 0.001

## Discussion

BSI includes prognostic information in addition to that obtained from clinical T stage, Gleason score and PSA. Patients with metastatic disease and BSI < 1 showed a 5-year probability of survival of 42% compared to 31% for those with BSI = 1 to 5 and 0% for those with BSI > 5. These findings show that a quantitative measurement of tumour burden in the skeleton can be used to risk-stratify patients more efficiently than only using M-staging based on evidence of the presence or absence of metastatic spread.

The added value of BSI was shown as an increase in C-index from 0.66 to 0.71 when adding BSI to a model including clinical T stage, Gleason score and PSA. This is in agreement with the results presented by Ulmert et al. [5], where the corresponding increase when adding BSI was of the same magnitude, but from a higher level (0.77 to 0.82). The higher values in the Ulmert study are likely to be due to the fact that death as a result of prostate cancer was used in that study, while all-cause mortality or overall survival was used in this one. The cause of death for the patients was not available in this retrospective study. Furthermore, the common primary end point in large clinical trials exploring the effects of drugs for prostate cancer patients is ‘overall survival’. This study showed the value of BSI in a homogenous group of prostate cancer patients, with those at high risk receiving primary hormonal therapy. In the Ulmert study, low-risk patients were also included and initial treatment was not adjusted for.

The sensitivity for metastatic disease of bone scans is regarded to be high, that is, the number of false-negative cases is low, but some of our M0 patients are probably falsely classified as M0. These patients might at least partly contribute to the higher mortality in the M0 population after 4 years in comparison with the age-matched control group. A follow-up bone scan to validate the BSI from the time of diagnosis could therefore be of value. Change in BSI over time can also contain important information in the M1 group. Dennis et al. recently showed that on-treatment change in BSI was associated with overall survival for patients receiving chemotherapy [4]. Their study also showed that changes in PSA were not associated with survival while adjusting for changes in BSI.

Recently, another study demonstrated the value of BSI in patients with castration-resistant prostate cancer [13]. In a group of 42 patients who underwent taxane-based chemotherapy, reduction of BSI after treatment was associated with longer overall survival. The findings of recent BSI studies show the value of this imaging biomarker in both at the time of diagnosis and at a later stage of the disease.

Based on the results of the present study, BSI can be considered as an informative predictor of patient survival in men with high-risk disease treated with hormonal therapy. BSI can also be of value in clinical trials as an inclusion criterion so that baseline prognosis in treatment and placebo groups can be matched.

The purpose of this study was to generate evidence regarding how BSI can be used to risk-stratify prostate cancer patients by applying an automated software to bone scans - the established method of evaluating skeletal metastases. Bone scans are widely used in both clinical routine and clinical trials, but the methodology has its shortcomings. The radiotracer locates not tumour cells but regions of the skeleton that are actively undergoing tissue repair - a well-known sign of tumour involvement. Future developments in diagnostic imaging may provide improved and more specific methods of analysing skeletal lesions, including three-dimensional methods such as SPECT/CT and PET/CT. At present, bone scan is the dominant clinical method for evaluation of skeletal metastases. Furthermore, prognostic studies based on large patient groups and long follow-up time are not currently available for the new modalities. This type of bone scan study, showing that imaging can be used as a biomarker, will be important in terms of gaining experience, which can also be of value when new imaging modalities become widely used.

This study was based on a fully automated software for calculation of tumour burden in patients with metastatic disease. With this technique, widespread use in clinical routine and clinical trials is possible. Operator-dependent subjectivity in interpretation of bone scans, which has proved to be substantial [14], is eliminated, and a reproducible analysis can be obtained at different hospitals. The software has proved to be capable of differentiating between malignant lesions and hotspots due to degenerative disease, e.g. by considering whether uptake in the shoulder regions is symmetrical. In this study, only obvious false lesions were manually corrected, e.g. a very large urinary bladder, a urinary catheter attached to a drainage bag or urine contamination. The quality of the software was assessed in a study by Sadik et al. [15]. This study showed that 35 physicians improved their sensitivity to metastatic disease from 78% to 88% using the software for automated analysis of bone scans.

## Conclusions

In conclusion, this study showed that BSI is strongly associated with overall survival in patients with high-risk prostate cancer receiving primary hormonal therapy and that BSI includes prognostic information in addition to clinical T stage, Gleason score and PSA. This study builds on previous studies and generates evidence that contributes to qualification of BSI as an imaging biomarker in prostate cancer patients. This biomarker has recently become automated and thus highly reproducible, eliminating operator-dependent subjectivity and providing prognostic information with a processing time < 10 s.

## Competing interests

Lars Edenbrandt and Mattias Ohlsson are shareholders in EXINI Diagnostics AB (Lund, Sweden), which provides software for Bone Scan Index calculations. Reza Kaboteh, Jan-Erik Damber, Peter Gjertsson, Peter Höglund and Milan Lomsky indicated no potential conflicts of interest.

## Authors’ contributions

RK and LE participated in the design of the study and in the analysis and interpretation of data, and drafted the manuscript. JED, PG and ML participated in the design of the study and in the analysis and interpretation of data. PH and MO carried out the analysis of data. All authors read and approved the final manuscript.
